# Trends in Deaths from Rheumatic Heart Disease in the Eastern Mediterranean Region: Burden and Challenges

**DOI:** 10.3390/jcdd5020032

**Published:** 2018-05-30

**Authors:** Azza M. A. M. Abul-Fadl, Maha M. Mourad, Alaa Ghamrawy, Ayah Ebada Sarhan

**Affiliations:** 1 Pediatric Department, Benha University, Benha 13511, Qalyibia, Egypt; 2 Pediatric Department, Pediatric Cardiology Unit, Cairo University, Cairo 11562, Egypt; mahammourad@hotmail.com; 3 Non communicable Disease Department, Ministry of Health and Population, Cairo 11562 Egypt; alaaelghamrawy@yahoo.com; 4 Department of Psychology, American University in Cairo, Fifth settlement, Cairo 11835, Egypt; ayahsar@aucegypt.edu

**Keywords:** rheumatic fever, rheumatic heart disease, trends, health services, deaths, global data

## Abstract

Rheumatic heart disease (RHD) is a preventable disease that is prevalent in developing regions of the world. Its eradication from most of the developed world indicates that this disease can be controlled and eliminated. **Aim:** To conduct an in-depth analysis of the trends and challenges of controlling RHD in the Eastern Mediterranean region (EMR). **Methodology:** Global data from the World Health Organization (WHO) data banks were retrieved for total deaths and age standardized death rate per 100,000 (ASDR) by age group, sex, and year (from 2000 to 2015). The data was compared with the five other WHO regions of the world. We also performed in-depth analysis by socio-economic groups in relation to other attributes in the region related to population growth, illiteracy, and nutritional status. Indicators of service delivery were correlated with ASDR from RHD. **Findings:** Prevalence of RHD in 2015 in the EMR region was one-third of that of the total deaths reported in the Asian and West Pacific regions. The total deaths for the region peaked twice: in early adulthood and again later in old age, and was higher in females than in males. There was a rising trend in deaths from RHD from 2000 to 2015. The highest total deaths were reported from Egypt, Pakistan, Iran, Afghanistan, and Yemen, representing 80% of the total death rates for the region (35,248). The highest ASDR was Afghanistan (27.5), followed by Yemen (18.78) and Egypt (15.59). The ASDR for RHD was highest in low income countries. It correlated highly, in all income groups, with anemia during pregnancy. **Conclusions:** Trends and patterns of deaths from RHD in the EMR have shifted to a later age group and are linked with poverty related to inequalities in development and service delivery for certain age groups and gender.

## 1. Introduction

Rheumatic heart disease (RHD) is a preventable disease that is linked with poverty, and is prevalent in developing regions of the world. Its eradication from most developed countries indicates that this disease can be controlled and eliminated.

The World Health Organization [1] was the first to release guidelines for the prevention and control of streptococcal disease and Rheumatic fever (RF) that have since been updated and adapted by many countries and regions around the world [2,3]. In Egypt in the 1960s, the Abdin and Eissa working group [4,5] established a link between streptococcal disease and RHD, and attributed it to poor living conditions, overcrowding, poor hygiene, and a lack of exposure to health education. They were among the first workers in the Egypt to report its high incidence in the tropics and its occurrence in children under-five years of age [5]. They identified 68 cases among children below 5 years of age, of whom 28 were below the age of 4, out of the one thousand children with RHD who attended their clinic for treatment. A systemic review of studies over the past 35 years in Egypt showed that prevalence of RHD has decreased since then by one fifth or less [6]. A recent analysis from the Global Burden of Disease 2015 study for the EMR region has shown that age-standardized death rates (per 100,000) (ASDR) from RHD have decreased from 9.1 in 1990s to 6.8 in 2015 (a 25% reduction) [7], although the number of cases overall has increased from 18,350 to 27,047 (a 47% increase). On the basis of such findings, the Nordet group [8], working with WHO, has lead awareness and community mobilization programs for the control of RHD in communities.

Recent data from the Global Burden of Diseases (GBD) Study [9] estimates that in 2015, RHD may have affected 31.4 million individuals worldwide [10]. Among these cases, 99% were attributable to endemic areas, mainly in low- and middle-income countries (LMICs), with an age-standardized prevalence of 4.44/100,000 population, compared with 3.4/100,000 population in non-endemic countries, the majority of which were high income [9].

The challenge of controlling and eradicating RHD is great, and although the disease has declined over past decades, recent data still indicate that the burden of streptococcal disease remains unchanged. In fact, the disease may be on the rise [11], especially with the introduction of echocardiography-based screening as a gold standard for early detection of RF and RHD [11,12]. Hence, the WHO and World Heart Federation (WHF) have called for a 25% reduction in mortality from cardiovascular causes, including RHD, by the year 2025 [13,14].

There is a paucity of data at the regional level in the Eastern Mediterranean region (EMR); this is an obstacle for undertaking adequate analyses of the status of RHD, and identifying the needs, priority areas, and patterns of distribution. Moreover, the recent adoption of a resolution on RF and RHD by the World Health Assembly on its seventy first meeting, in Geneva, calls for action on part of the governments to make effective plans for its control and eradication (WHO/WHA, 2018). Hence, the aim of this study is to build on the findings of the Global Burden of Disease Study (2015), with a focus on RHD prevalence in the EMR, and also to analyze the trends in total deaths from RHD and review challenges facing its control and eradication in EMR countries. 

## 2. Methods

Global data from the World Health Organization (WHO) data banks were compiled and analyzed for deaths from RHD in the EMR by gender and other factors from 2000 to 2015.

Data for the individual countries were retrieved from the World Bank and WHO (http://www.worldlifeexpectancy.com). The site uses data from these primary sources: WHO, World Bank, UNESCO, CIA, and individual country databases for global health and causes of death.

Data for service delivery and demographic data were taken in 2017 from the WHO-EMR Framework of Health Information Systems and core indicators for monitoring health situations and health system performance. The data represented surveys from 2014 and 2015.

The data was compiled in Excel spreadsheets, and was statistically analyzed using standard methods.

The countries were categorized by income level into three groups:

- High income countries (HIC): Bahrain, Kuwait, Qatar, Saudi Arabia, UAE, and Oman.
- Middle income countries (MIC): Egypt, Jordan, Iran, Iraq, Lebanon, Libya, Morocco, Syria, Tunisia, and Palestine.
- Low income countries (LIC): Afghanistan, Djibouti, Pakistan, Somalia, Sudan, and Yemen.

**Ethical considerations**: The work of this paper was in compliance with the ethical principles of the Helsinki Declaration (1964).

**Statistical Analysis**: The collected data were organized and tabulated first on Excel speadsheets for initial statistical analysis. The relevant data were further analyzed using SPSS version 20 (SPSS Inc.; Chicago, IL, USA, running on IBM compatible computer with Microsoft Windows 7 Operating System. The Student *t*-test (for parametric data) and Mann-Whitney U test (for non-parametric data) were used for comparison of two distinct groups. Analysis of variance with a post hoc Bonferroni test (for parametric data) and Kruskal-Wallis test, followed by a Mann-Whitney U test (for non-parametric data), were used to compare groups. Categorical variables were compared using Chi square and Fisher exact tests. The Spearman rank correlation coefficient was used to measure the strength and direction of linear relationship between two variables. The level of significance cut off was set at *p* < 0.05. 

## 3. Results

**Figure 1:**
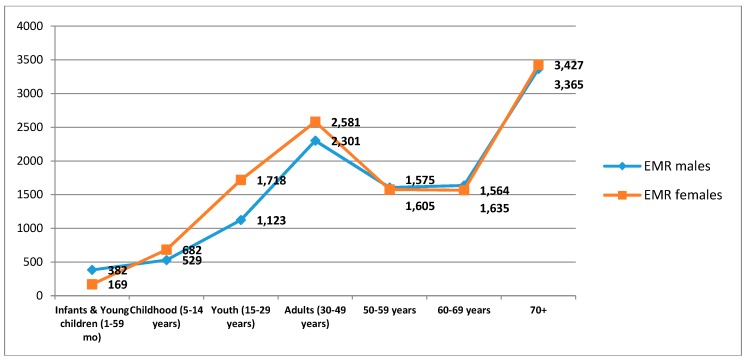
Trends in total deaths (Y axis) from Rheumatic Heart Disease by age and sex in the Eastern Mediterraenan Region (EMR).

The findings presented in the Figure 1 show that the pattern of RHD increased in early adulthood (30–49 years), and then peaked again in the elderly after 70 years of age in both males and females. The pattern of deaths demonstrated a double peak that was slightly higher in females than males, exhibiting a more prominent peak in late adolescence and early adulthood, probably reflecting the increased risk of death during pregnancy and labor.

**Figure 2:**
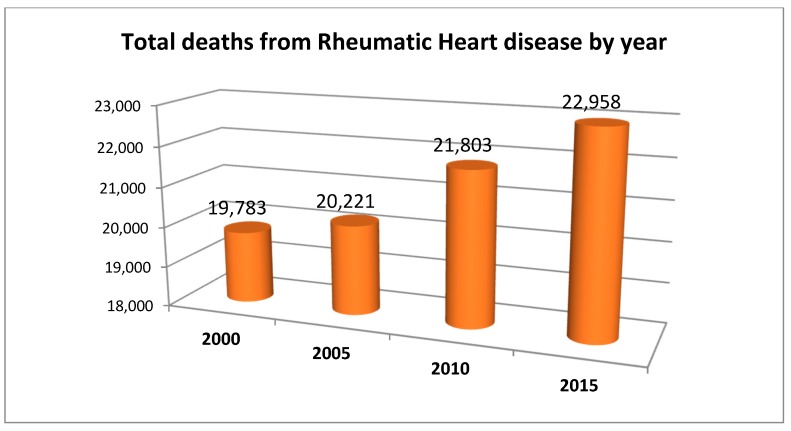
Comparison of the trends in total deaths from rheumatic heart disease in the Eastern Mediterranean region from the year 2000 to 2015.

Figure 2 shows that there was a rising trend in the total deaths from RHD from the years 2000 to 2015. This was more apparent between 2005 and 2010, as the percentage change was highest (8%) compared to the 2% change between 2000 and 2005 and the 5% change between 2010 and 2015. Although data of total deaths may be misleading (as they depend on total population and population density), they nonetheless give some insight into the magnitude of the problem of RHD in the region.

**Figure 3:**
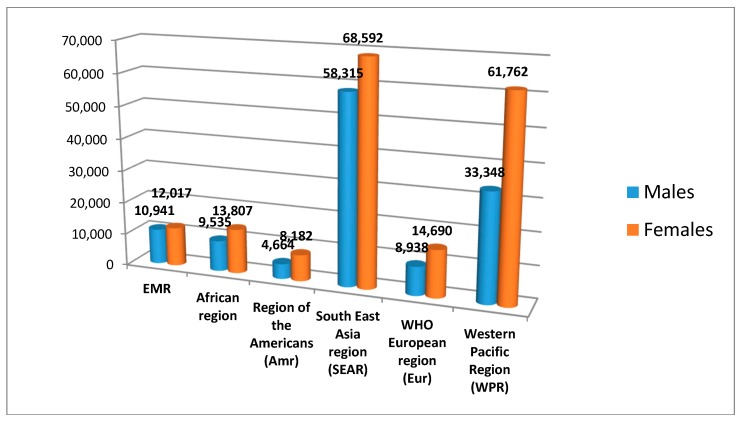
Comparison of the prevalence of Rheumatic heart disease in the Eastern Mediterranean region with other regions in the world in 2015.

Figure 3 compares the total deaths from RHD in the EMR with the other regions. It shows that the total deaths from RHD in South East Asia region (SEAR) was 4.3 times higher for males and 4.7 times higher for females than that observed in the EMR. Also, total deaths in the West Pacific region (WPR) were two times higher among males, and four times higher among females, compared to EMR region rates. The African region had the same death toll as the EMR. This could be confounded by differences in under detection and under reporting in the EMR and African region, thereby underestimating the actual prevalence of the disease.

**Figure 4:**
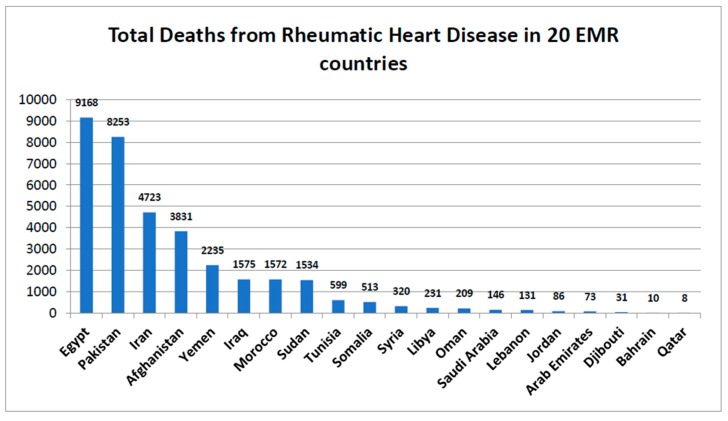
Total deaths from Rheumatic heart disease for the 20 countries with reported data in the Eastern Mediterranean region (Data source (www.worldlifeexpectancy.com), based on WHO data in 2014).

Figure 4 shows the total deaths from RHD at national levels. This data was obtained from another source (the World Bank and WHO, 2014). The total deaths for EMR from this source showed a higher burden of deaths (35,248) than the data of Global Burden of Disease Study (22,956), although they represented a one year difference (2014 and 2015 respectively). Total deaths by country was highest in Afghanistan (9168) and lowest in Qatar (8). Total deaths reported from Egypt, Pakistan, Iran, Afghanistan, and Yemen represented 80% of the total deaths for the region (35,248).

**Figure 5:**
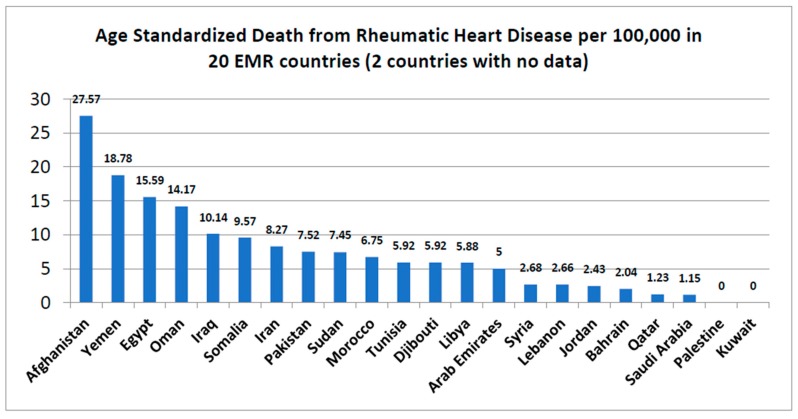
Age standardized deaths per 100,000 from Rheumatic Heart Disease for the 20 countries of the Eastern Mediterranean region. Source: (www.worldlifeexpectancy.com) Data source: published by WHO, 2014.

Figure 5 illustrates the prevalence by age standardized death rate (ASDR) per 100,000 in 20 out of the 22 countries in the region. No data were reported from Palestine and Kuwait. The highest age standardized death rate per 100,000 was Afghanistan (27.5), followed by Yemen (18.78) and Egypt (15.59); the lowest was in Saudi Arabia (1.15).

Table 1 shows the mean ASDR for RHD per 100,000. By income group, ASDR for RHD was highest in the LIC (12.80 ± 8.58) compared to MIC (6.0 ± 4.54) and HIC (3.93 ± 5.29). Hence, ASDR increased with decreasing levels of income in the countries under study, reflecting the importance of improving social conditions for preventing the disease.

Table 1 shows that correlation coefficients of ASDR from RHD with selected social indicators were significant for anemia during pregnancy (r0.74), mortality from exposure household and ambient air pollution (MAAP) (r0.6), literacy (r-0.58), and total fertility rates (TFR) (r0.57). It was also present, but not statistically significant, for maternal mortality ratio (MMR) (r0.49) and low birth weight (LBW) (r0.46). 

Analysis by income groups showed that in high-income countries, there was significant correlation of ASDR per 100,000 from RHD by country with total literacy (r0.87), female literacy (r0.84), and the presence of anemia during pregnancy (r0.83), at *p* < 0.05. In middle income countries, there were significant correlations of ASDR per 100,000 from RHD by country with mortality from ambient air pollution (r0.81) and anemia during pregnancy (r0.74), at *p* < 0.05 and 0.001 respectively. In low-income countries, there was a moderate correlation of ASDR per 100,000 from RHD by country with anemia during pregnancy (r0.52), at *p* < 0.05 as shown in Table 1.

Table 1 also demonstrates associations between health care management of services and ASDR per 100,000 from RHD, as follows. For all countries, ASDR per 100,000 correlated with nursing and midwifery per 1000 population (r-0.48), at *p* < 0.05, while correlating negatively with per capita total expenditure on health (r-0.49), at *p* < 0.05, and out-of-pocket expenditure as a percentage of total health expenditure (r0.53), at *p* < 0.05. The correlative studies by income group for the same indicators were as follows. In high-income countries, it correlated with physician and nursing per 1000 population (r-0.84 and r-0.76), at *p* < 0.05 and *p* < 0.05 respectively, and with primary health care facilities per 10,000 population (r0.78), at *p* < 0.05. Whereas in middle-income countries, Age Standardized Death rate per 100,000 from RHD correlated negatively with physician per 1000 population (r-0.61), at *p* < 0.05, and per capita total expenditure on health care (r-0.69), at *p* < 0.05. In low-income countries, Age Standardized Death rate per 100,000 from RHD correlated with physician per 1000 population (r-0.66), at *p* < 0.05.

## 4. Discussion

Our study shows that the total deaths from RHD are not declining possibly related to improved detection and reporting. The pattern of the burden of death from RHD in the EMR countries is moving to a higher age group compared to the patterns reported in the past century [4,5,6]. Late adolescence and early adulthood are mostly at risk of death from RHD, possibly due to the drop-out from health care, and gaps in adolescent health care services in these age groups, particularly in middle- and low-income countries. The higher burden in females reflects increased risk from pregnancy and childbirth.

The differences in the total deaths between regions probably reflect under detection and under reporting, rather than de facto lower prevalence in the EMR and Afro regions compared with the SEAR and WPR. Still, the burden was shown to be in the range of 3–5 times, which is a significant difference, and more in-depth analysis is needed.

Our findings run in parallel with those of the EMR team of Global Burden of Disease Study Group [7], who presented an elegant study of the overall burden of deaths from cardiovascular diseases (CVD) in the EMR, including deaths from RHD and disability related life years (DALYS) from this disease. They highlighted the decrease in prevalence of the disease from 1990 to 2015 by one third, but did not perform a micro-analysis of the period from 2000 and 2015. They emphasized that cardiovascular diseases, particularly ischemic heart disease, is the leading cause of mortality in the region [7]. We argue that although death from RHD represents a minor portion of overall death from CVD, the DALYs for RHD are particularly high because of the wider age range involved, starting from early childhood and extending throughout the remainder of life. The prolonged period of disability these individuals suffer as children and adults influences their quality of life by decreasing their opportunities, increasing their poverty, and decreasing their life expectancy [7].

A recent study [8] estimated that there were 319,400 deaths in 2015. They reported that global age-standardized mortality from RHD decreased from 9.2 deaths per 100,000 population in 1990 to 4.8 deaths per 100,000 population in 2015, showing a decrease of 47.8%. They reported that the largest number of deaths occurred in East and South Asia, which is in agreement with the findings in this study. However our study does not show the decrease in death from RHD from 2000 to 2015 that was described in their study. This decrease may have been true for other regions of the world and for the HIC in EMR, but not for the LIC and MIC, as shown by the differences of ASDR by income group in this study.

The Watkins team estimated that the global prevalence of RHD was 33.4 million cases worldwide, which is really only the tip of the iceberg, as expressed by Marijon [10], and is more than twice that calculated by a systematic evaluation of the literature [15]. The estimate was based on modeling due to a lack of data; for instance, 15 out of 53 countries in Africa lack primary sources of data on deaths from RHD [16].

In this analysis, EMR reported deaths from RHD totaled 22,980. The latter is probably an underestimation, since, in the Watkins study, the estimate of deaths in Pakistan alone was 18,900. Also our data estimate the number of deaths from SEAR to be 126,907, while the Watkins study group [16] estimated that the numbers of deaths due to RHD in India and China alone (119,100 and 72,600 deaths respectively) were much higher than those reported by the WHO for the region. Other contributors [9] to the journal in which the study of the Watkins team was published argue that the calculations may be an underestimate. They argue that by limiting heart failure as the main underlying cause of death, their estimates may have suppressed other causes of death as causes or contributing factors to RHD [17], such as atrial fibrillation, endocarditis, severe pulmonary venous hypertension, stroke, failure of anticoagulation therapy, and decompensation associated with pregnancy [18].

The Watkins study reported that a high ASDR, more than 10 deaths per 100,000 population from RHD, was identified in Pakistan in the EMR, Central African Republic and Lesotho in Africa, India, from Asia, and Solomon Islands, Papua New Guinea, Kiribati, Vanuatu, Fiji, Federated States of Micronesia, and the Marshall Islands from West Pacific regions [9]. Other authors pursue the argument that the global community cannot be complacent on the issue of RHD control, as RHD still prevails in pockets of low socioeconomic activity, and among immigrants even in developed countries [19,20,21].

In this study, we found high correlations of ASDR from RHD with social indicators such as literacy and TFR. RHD is a disease of poverty, and primordial prevention plays a significant role in its prevention. The disease has been eradicated in regions by simply improving living conditions, personal hygiene, and access to clean water, as well as increasing awareness and education [8]. The crowding index, which is closely linked to TFR, has been closely linked with higher prevalence of streptococcal disease [4,5,6]. This might explain the high correlation with the limited social indicators reported in our study, but nonetheless warrants further investigation [16].

Furthermore we found some moderate correlations with MMR and LBW which again reflect the higher prevalence and higher burden among females. This is in agreement with the findings of other workers [15], who reported that the disease is more prevalent among females. Possible causes include late detection, exposure to social discrimination, stigma from the disease interfering with prospects of marriage (especially in females living in socially deprived areas), illiteracy, and disempowerment [15,16].

In this study we showed correlations of ASDR from RHD and service management issues. The Australian “continuous quality approach” [22] for modeling service delivery in high-risk communities has shown itself to be effective [23]. It emphasizes the need for improving communication in the delivery of services [24]. However it is important to emphasize that improving service delivery alone cannot prevent the disease; it is more important to improve the living conditions for patients. This requires in-depth needs assessments, involving multifaceted interventions and mapping of resources and stakeholders, in order to identify the best approaches to deliver community-based services which are tailored to the needs of sustained development [25,26].

The primary health care approach is ideal for the prevention and control of RHD, as it represents the front line for detection and adequate treatment of sore throats for primary prevention [27], ensuring compliance for secondary prevention and referral for efficient management of cases at tertiary levels [28]. To do so, it requires intensive training and capacity building of primary health care and managerial staff, in order to manage efficient registries and referral systems [29]. With problems of understaffing, rapid turnover, and over-burdened, poorly paid, and inadequate monitoring and evaluation, many cases can be missed or can drop out of the system, increasing the burden of disability, which was estimated to be 1430 DALYS in 2010 [30], the equivalent of one quarter of the Global DALYS of cancer cases, leading to high expenditure with poor survival rates in complicated cases [14].

Mortality rates have been underestimated for decades. In the global systemic review of 2005, the burden of RHD estimates comprised 15.6 million cases, 282,000 incident cases, and 233,000 deaths annually [15,31]. A more recent analysis calculated a much higher figure of 314,000. The actual burden may still be higher, and methods to improve such estimates have been considered by other workers [32]. Our findings of differential data between WHO global data banks, other data banks, and national reporting, indicate that the burden of the disease may continue to represent a challenge [30].

RHD is a marginalized, neglected, poorly capitalized and poorly-addressed disease that continues to face barriers related to inequality and poor accessibility and dismantlement of health services [33]. The challenges related to controlling RHD are related to under detection, under reporting, poor compliance, poor access to care, poor prognosis, poor postoperative care, poor registration systems, and lack of equitable access to gold standard screening methods, lack of culturally sensitive adolescent health services and inadequate school (and university) health care systems [34,35]. In developing countries, where referral systems and continuity of care is lacking, the problem of RHD prevention and control will prevail, unless reforms based on models that ensure continuity of care and equity are enacted by governments [36].

From a developmental perspective, RHD is a disease of poverty, inequitable distribution of resources, and social inequality [37,38]. For instance, females in this study had a higher prevalence of death from RHD than males in all regions. This can be explained by social factors, as discussed earlier, but also by poor access to health care for early detection, lack of resources, and lack of awareness and education about the necessity of access to contraception. Increased mortality really begins at puberty and menstruation. It is at this time that females shift toward immune TH2 pathways making them more vulnerable to the immunological changes accompanying the development and progression of RHD [5]. The exacerbation of the underlying immune mediated responses to RHD is likely to explain why women have more complications than men at this age.

The REMEDY group [39] reported that females were more affected. This was a prospective registry study that enrolled 3343 patients with RHD from 25 hospitals in 12 African countries, and also included countries from the Asia as India. Among the 1825 women of childbearing age (12–51 years), only 3.6% (*n* = 65) were on contraception. One third of the cases indicated to receive anticoagulants were not receiving them, and only one third of the cases which were on anticoagulants had a therapeutic international normalized ratio. The utilization of valvuloplasty and valve surgery was higher in upper-middle-income, compared with lower-income, countries [28]. Such findings can explain the higher prevalence of deaths among females, particularly those living in poverty. The authors concluded that female patients are the most vulnerable population, due to suboptimal utilization of secondary antibiotic prophylaxis, oral anti-coagulation, and poor access to contraception and timely surgical interventions [39].

Several investigators [16] have discussed the fact that the many challenges to the prevention and control of RF/RHD can be helped by estimating the true global burden of the disease. They emphasize that although registries with good quality of data are important, this has been limited to countries such as Australia, New Zealand, Pacific Islands, South Africa, and Tunisia, and is lacking in many other countries inflicted with this disease. Screening methods are archaic and need to be updated [6,40]. There is a need for capacity building of physicians and primary health care providers to notify, correctly report positive diagnoses and manage RF/RHD prevention and control programs by developing short and long term courses, qualifications and degree programs to be adopted by universities in developed and developing countries [36].

Although primordial prevention, followed by improving service delivery, is the primary control strategy for the control of RHD, poverty and inequity impede program inputs. For instance, countries with poor economies in many settings in EMR and African countries lack the consistent supply of quality-assured benzathine benzylpenicillin for primary prevention and secondary prophylaxis [41]. This is even happening in some pockets in developed countries [42]. Manufacturers in such countries fail to supply these drugs because of the price inflations that impede their manufacturing and supply at low cost for the poor. Governments need to network with international manufacturers to upgrade these medicines and make them available through subsidization for the poor and needy populations affected by the disease [37,43,44].

Another major barrier is that governments are overwhelmed with other priorities, and do not see RHD as a priority, as it predominantly affects poor and marginalized communities. Hence, the lack of advocacy and persistent marginalization of RHD control programs by governments, coupled by poor funding and budget allocation of resources for the program, make the health system unable to implement efficient services for early detection, referral, follow-up, and provision of essential care, particularly in low-income settings [37,38].

Advocacy can be strengthened by finding synergy between RHD, and established high-priority targets in global health could help RHD gain a stronger foothold. For example, the sustainable development agenda and the recent global focus on NCD prevention have both provided a new platform for RHD advocacy [45,46,47]. Moreover, in June 2017, the Executive Board of the WHO recommended a resolution (expected to be adopted at the 71st World Health Assembly (WHA) in 2018) on “rheumatic fever and RHD” [45].

Because RHD is a disease of poverty, its persistence in poor countries handicaps and aggravates the economic situation in these countries. RHD starts in early childhood, and thereby increases the DALYs which adversely influence the economy [37]. Globally, RHD is the fifth leading cause of cardiovascular-related mortality, and the sixth leading cause of cardiovascular-related disability. The combination of years of life lost (premature deaths) and years lived with disability, expressed as DALYs, is 10.5 million/year for RHD [44,47].

The Addis Ababa communiqué, as follows, has since been endorsed by heads of state of the African Union and EMR countries: (1) create prospective disease registers at sentinel sites in affected countries to measure disease burden and track progress towards a reduction of mortality by 25% by the year 2025, (2) ensure an adequate supply of high-quality benzathine penicillin for the primary and secondary prevention of ARF/RHD, (3) improve access to reproductive health services for women with RHD and other non-communicable diseases (NCD), (4) decentralize technical expertise and technology for diagnosing and managing ARF and RHD (including ultrasound of the heart), (5) establish national and regional centers of excellence for essential cardiac surgery for the treatment of affected patients and training of cardiovascular practitioners of the future, (6) initiate national multi-sectorial RHD programs within the NCD control programs of affected countries, and (7) foster international partnerships with multinational organizations’ for resource mobilization, monitoring and evaluation of the program to end RHD in Africa [48]. Plans are underway to implement a roadmap to end ARF and RHD in Africa in our lifetime, and change trends and outcomes of the disease in our region and around the world [43,44,49,50].

## 5. Conclusions

We emphasize the important role of social determinants and the importance for primordial preventive strategies as key to the eradication of RHD. This should include improving education opportunities, especially for females, and access to birth control services, improving living conditions and intensifying awareness campaigns by using the underutilized social media eHealth platforms of networking and health promotion. Primary health care in the region should aim at reforming delivery of its services, particularly for adolescents, thereby reducing inequity, exclusion, and social disparities in health, so as to achieve universal coverage. The health services should be organized around people’s needs and expectations, and this requires service delivery reforms. RHD should become a priority for governments, and an index for human development. This requires a collaborative model of policy dialogue, increased stakeholder participation, and pressure from international agencies. Finally, the challenge to eradicate RHD in the EMR remains with low- and middle-income countries in the region, i.e., 70% of the countries in the region, and over 90% of its total population. These communities suffer from chronic emergencies and political instability [20], which increase the burden of poverty and rates of integration in primary health care provisions, and thereby continue to make RHD a potential risk for increased disability and poor development. The World Health Assembly resolution for RF and RHD on its 71st meeting is expected to bring hope for renewed efforts to control and eliminate Rheumatic fever and Rheumatic heart disease globally [44].

## Figures and Tables

**Table 1:** Correlative studies between Age Standardized Death Rate (ASDR) per 100,000 from Rheumatic Heart Disease (RHD) with selected social demographic indicators.

Indicators	Total (20)	HIC (5)	MIC (9)	LIC (6)
Age Standardized Death Rate (ASDR) from RHD (mean ± SDs)	7.31 ± 6.76	3.93 ± 5.29	6.0 ± 4.54	12.80 ± 8.58
**Correlative studies of ASDR with selected socio-demographic indicators**				
Literacy (total)	r-0.58 *	r-0.877 *	r-0.877 *	r-0.084
Literacy (in females)	r-0.51 *	r-0.836 *	r-0.537 *	r-0.328
Total fertility rate (TFR)	r0.57	r0.89 *	r0.08	r0.27
Deaths from household & ambient air pollution (MAAP)	r0.6 *	r-0.11	r0.81 *	r0.31
Anaemia during Pregnancy	r0.74 *	r0.832 *	r0.83 *	r0.52 *
Maternal mortality ratio (MMR)	r0.49	r0.5 *	r-0.07	r0.19
Low birth weight (LBW)	r0.46	r0.22	r-0.05	r0.07
**Correlative studies of ASDR with selected health service indicators**				
Physician per 1000 population	r-0.43	r-0.84 *	r-0.61 *	r0.66 *
Nursing and midwifery per 1000 population	r-0.48 *	r-0.76 *	r-0.15	r0.13
Per capita total expenditure on health (US$ exchange rate)	r-0.49 *	r-0.69 *	r-0.28	r-0.29
Out of pocket expenditure as a percentage of total health expenditure	r0.53 *	r-0.42	r0.46	r0.40
Primary Health Care facilities per 10,000 population	r-0.28	r0.798 *	r-0.39	r-0.32
Hospital beds per 10,000 population	r-0.10	r-0.4	r-0.12	r-0.4

*p*-Value (level of significance) *p* < 0.05 *.

## References

[1] World Health Organization (1954). Rheumatic Diseases: First Report of the Expert Committee.

[2] RHD Australia (ARF/RHD Writing Group), National Heart Foundation of Australia and the Cardiac Society of Australia and New Zealand (2012). Australian Guideline for Prevention, Diagnosis and Management of Acute Rheumatic Fever and Rheumatic Heart Disease.

[3] Heart Foundation of New Zealand (2014). Group A Streptococcal Sore Throat Management Guideline.

[4] Abdin Z., Eissa A.M. (1965). Rheumatic fever and rheumatic heart disease in children below the age of 5 years in the tropics. Ann. Rheum. Dis..

[5] Toor D., Sharma N. (2018). T cell subsets: An integral component in pathogenesis of rheumatic heart disease. Immunol. Res..

[6] Abul-Fadl A.M.A., Mourad M.K. A systemic review of screening methods for rheumatic heart disease in Egypt: Potential role of echocardiography for community based surveys. Proceedings of the Euro Eco Imaging 2014 18th Annual Meeting of the European Association of Cardiovascular Imaging.

[7] Mokdad A.H. (2017). Burden of cardiovascular disease in the Eastern Mediterranean region 1990–2015: Findings from the Global Burden of Disease 2015 study. Int. J. Public Health.

[8] Nordet P., Lopez R., Dueñas A., Sarmiento L. (2008). Prevention and control of rheumatic fever and rheumatic heart disease: The Cuban experience (1986–1996–2002). Cardiovasc. J. Afr..

[9] Watkin D., Johnson C.A., Colquhoun S.M., Karthikeyan G., Beaton A., Bukhman G., Forouzanfar M.H., Longenecker C., Mayosi B.M., Mensah G.A. (2017). Global, Regional, and National Burden of Rheumatic Heart Disease, 1990–2015. N. Engl. J. Med..

[10] Marijon E., Celermajer D.S., Jouven X. (2017). Rheumatic heart disease-an iceberg in tropical waters. N. Engl. J. Med..

[11] Marijon E., Ou P., Celermajer D.S., Mocumbi A.O., Jani D., Paquet C., Jacob S., Sidi D., Jouven X. (2007). Prevalence of rheumatic heart disease detected by echocardiographic screening. N. Engl. J. Med..

[12] Nascimento B.R., Beaton A.Z., Nunes M.C., Diamantino A.C., Carmo G.A., Oliveira K.K., Oliveira C.M., Meira Z.M., Castilho S.R., Lopes E. (2016). Echocardiographic prevalence of rheumatic heart disease in Brazilian schoolchildren: Data from the PROVAR study. Int. J. Cardiol..

[13] World Health Organization (2013). Global Action Plan for the Prevention and Control of Noncommunicable Diseases 2013–2020.

[14] Remenyi B., Carapetis J., Wyber R., Taubert K., Mayosi B.M. (2013). Position statement of the World Heart Federation on the prevention and control of rheumatic heart disease. Nat. Rev. Cardiol..

[15] Carapetis J.R., Steer A.C., Mulholland E.K., Weber M. (2005). The global burden of group A streptococcal diseases. Lancet Infect. Dis..

[16] Dougherty S., Beaton A., Nascimento B.R., Zühlke L.J., Khorsandi M., Wilson N. (2018). Prevention and control of rheumatic heart disease: Overcoming core challenges in resource-poor environments. Ann. Pediatr. Cardiol..

[B17-jcdd-05-00032] 17.Sohrabi, B.; Ranjbar, A. Correspondence to “Global Burden of Rheumatic Heart Disease”.

[B18-jcdd-05-00032] 18.Jain, Y.; Juneja, R.; Patil, S. Correspondence to “Global Burden of Rheumatic Heart Disease”.

[B19-jcdd-05-00032] 19.Dickinson, J.A.; Johnston, I. Correspondence to “Global Burden of Rheumatic Heart Disease”.

[20] Rossi G., Lee V.S.W. (2016). Call for preventive care for rheumatic heart disease in refugee children. BMJ.

[21] De Maio G., Lupiz M., Condemi F., Pagano A., Al-Rousan A., Rossi G. (2016). Screening for Rheumatic Heart Disease in Refugee Children in Europe—MSF Leads, Will Others Please Follow?.

[22] Belton S., Kruske S., Pulver L.J., Sherwood J., Tune K., Carapetis J., Vaughan G., Peek M., McLintock C., Sullivan E. (2017). Rheumatic heart disease in pregnancy: How can health services adapt to the needs of Indigenous women? A qualitative study. Aust. N. Z. J. Obstet. Gynaecol..

[23] Saleh S., Alameddine M., Mourad Y., Natafg N. (2015). Quality of care in primary health care settings in the Eastern Mediterranean region: A systematic review of the literature. Int. J. Qual. Health Care.

[24] Ralph A., Fittock M., Schultz R., Thompson D., Dowden M., Clemens T., Parnaby M.G., Clark M., McDonald M.I., Edwards K.N. (2013). Improvement in rheumatic fever and rheumatic heart disease management and prevention using a health-centre based continuous quality improvement approach. BMC Health Serv. Res..

[25] Moloi A.H., Watkins D., Engel M.E., Mall S., Zühlke L. (2016). Epidemiology, health systems and stakeholders in rheumatic heart disease in Africa: A systematic review protocol. BMJ Open.

[26] El-Jardali F., Saleh S., Khodor R., Abu Al Rub R., Arfa C., Ben Romdhane H., Hamadeh R.R. (2015). An institutional approach to support the conduct and use of health policy and systems research: The Nodal Institute in the Eastern Mediterranean Region. Health Res. Policy Syst..

[27] Bergmark R., Bergmark B., Blander J., Fataki M., Janabi M. (2010). Burden of disease and barriers to the diagnosis and treatment of group a beta-hemolytic streptococcal pharyngitis for the prevention of rheumatic heart disease in Dar Es Salaam, Tanzania. Pediatr. Infect. Dis. J..

[28] Zühlke L., Engel M.E., Karthikeyan G., Rangarajan S., Mackie P., Cupido B., Mauff K., Islam S., Joachim A., Daniels R. (2015). Characteristics, complications, and gaps in evidence-based interventions in rheumatic heart disease: The Global Rheumatic Heart Disease Registry (the REMEDY study). Eur. Heart J..

[29] McDonald M., Brown A., Noonan S., Carapetis J.R. (2005). Preventing recurrent rheumatic fever: The role of register based programmes. Heart.

[30] Wyber R., Zühlke L., Carapetis J. (2014). The case for global investment in rheumatic heart-disease control. Bull. World Health Organ..

[31] Carapetis J. (2007). Rheumatic heart disease in developing countries. N. Engl. J. Med..

[32] Krahwinkel W., Schuler E., Liebetrau M., Meier-Hellmann A., Zacher J., Kuhlen R. (2016). For the Helios Medical Board and Helios Working Group on Peer Reviewing. The effect of peer review on mortality rates. Int. J. Qual. Health Care.

[33] Katzenellenbogen J.M., Ralph A.P., Wyber R., Carapetis J.R. (2017). Rheumatic heart disease: Infectious disease origin, chronic care approach. BMC Health Serv. Res..

[34] Kronfol N.M. (2012). Delivery of health services in Arab countries: A review. East. Mediterr. Health J..

[35] Nkgudi B., Robertson K.A., Volmink J., Mayosi B.M. (2006). Notification of rheumatic fever in South Africa–Evidence for underreporting by health care professionals and administrators. S. Afr. Med. J..

[36] Chamberlain-Salaun J., Mills J., Kevat P.M., Rémond M.G.W., Maguire G.P. (2016). Sharing success–understanding barriers and enablers to secondary prophylaxis delivery for rheumatic fever and rheumatic heart disease. BMC Cardiovasc. Disord..

[37] Wagstaff A. (2002). Poverty and health sector inequalities. Bull. World Health Organ..

[38] Peters D.H., Garg A., Bloom G., Walker D.G., Brieger W.R., Rahman M.H. (2008). Poverty and access to health care in developing countries. Ann. N. Y Acad. Sci..

[39] Zühlke L., Karthikeyan G., Engel M.E., Rangarajan S., Mackie P., Cupido-Katya Mauff B., Islam S., Daniels R., Francis V., Ogendo S. (2016). Clinical outcomes in 3343 children and adults with rheumatic heart disease from 14 low- and middle-income countries: Two-year follow-up of the global rheumatic heart disease registry (the REMEDY study). Circulation.

[40] Carapetis J.R., Hardy M., Fakakovikaetau T., Taib R., Wilkinson L., Penny D.J., Steer A.C. (2008). Evaluation of a screening protocol using auscultation and portable echocardiography to detect asymptomatic rheumatic heart disease in Tongan school children. Nat. Clin. Pract. Cardiovasc. Med..

[41] Guilfoyle J. (2015). Out of sight, out of mind. Can. Fam. Physician.

[42] Johnston I., Gittens C., Dickinson J.A. Pharyngitis complications in North America in the 21st century. Proceedings of the Third Preventing Overdiagnosis Conference.

[43] World Health Organization (WHO) (2017). Rheumatic Heart Disease.

[44] World Health Organization (WHO) (2017). Rheumatic Fever and Rheumatic Heart Disease.

[45] Murray C.J., Vos T., Lozano R., Naghavi M., Flaxman A.D., Michaud C., Ezzati M., Shibuya K., Salomon J.A., Abdalla S. (2012). Disability-adjusted life years (DALYs) for 291 diseases and injuries in 21 regions, 1990-2010: A systematic analysis for the Global Burden of Disease Study 2010. Lancet.

[46] Yusuf S., Wood D., Ralston J., Reddy K.S. (2015). The World Heart Federation’s vision for worldwide cardiovascular disease prevention. Lancet.

[47] Palafox B., Mocumbi A.O., Kumar R.K., Ali S.K.M., Kennedy E., Haileamlak A., Watkins D., Petricca K., Wyber R., Timeon P. (2017). The WHF Roadmap for Reducing CV Morbidity and Mortality through Prevention and Control of RHD. Glob. Heart..

[48] Watkins D., Zuhlke L., Engel M., Daniels R., Francis V., Shaboodien G., Kango M., Abul-Fadl A., Adeoye A., Ali S. (2016). Seven key actions to eradicate rheumatic heart disease in Africa: The Addis Ababa communiqué. Cardiovasc. J. Afr..

[49] Sorour K. (2014). Rheumatic heart disease in Egypt: Gloomy past and promising future. Egypt. Heart J..

[50] Sriha Belguith A., Koubaa Abdelkafi A., El Mhamdi S., Ben Fredj M., Abroug H., Ben Salah A., Bouanene I., Hassine F., Amara A., Bhiri S. (2017). Rheumatic heart disease in a developing country: Incidence and trend (Monastir; Tunisia: 2000–2013). Int. J. Cardiol..

